# 4.E. Workshop: The role of National Public Health Institutes and IANPHI as Key Climate Actors

**DOI:** 10.1093/eurpub/ckac129.223

**Published:** 2022-10-25

**Authors:** Organised by: Robert Koch Institute, IANPHI (France), Chair persons: Angela Fehr (Germany), Sébastien Denys (France)

## Abstract

Climate change is arguably the greatest threat to population health worldwide. Urgent action is needed to mitigate and adapt to the impacts. National public health institutes (NPHIs) are key actors in preventing illness and improving the health and wellbeing of their populations. They therefore have a crucial role to play in addressing climate change and making it central to their agendas. They can contribute to a better understanding of how climate change affects health, translate this knowledge into policy advice, and identify health co-benefits and possible harms of mitigation and adaptation measures to support the implementation of healthy measures within and beyond the health sector. NPHIs are also responsible for reducing their own footprint through “greening” and can role model and support other organisations in this regard. The International Association of National Public Health Institutes (IANPHI) recognises the threat that climate change poses and, to this end, published the “IANPHI Roadmap for Action on Health and Climate Change” in November 2021. This roadmap highlights the essential role of NPHIs’ as “key climate actors”. NPHIs are at different stages of progress on climate change and health, and there is therefore need and opportunity to learn from best practices and to liaise and work together to address shared challenges. It is also essential that NPHIs have an ongoing collaboration with other actors, such as academics, civil society, NGOs and other non-state actors, within and beyond the health sector, in order to learn from other perspectives and sources of knowledge and to ensure coherence and joint action on climate change and health across the system. This workshop therefore aims to promote discussion and exchange and to raise the visibility of NPHIs as key climate actors in order to strengthen their individual and shared contribution. The workshop will also ensure that the IANPHI Roadmap remains front of mind a year after publication. It will also give the opportunity to NPHIs to share their progress since then, as well as promote coherence and exchange in their continued efforts. The workshop will start with a presentation of the IANPHI Roadmap, followed by three examples from NPHIs on their progress on climate change strategy development and implementation. It will then be followed by an interactive session and discussion on NPHIs’ priorities in this area, on how NPHIs and other actors can best exchange and work together, and on how IANPHI as a global network of NPHIs can promote and support NPHI and stakeholder cooperation. The presentations are coherent together in offering the Roadmap's vision as well as a set of diverse examples that illustrate the role of NPHIs on climate change and health in practice.

**Key messages:**

• Emphasis of the important role that NPHIs have in addressing climate change and health, as well as the value of their partnerships and intersectoral working, which can be supported by IANPHI.

• Promotion of discussion and new ideas between the presenters and the audience on how to ensure health is at the forefront when tackling climate change.

